# Synergistic Induction of Apoptosis in Primary B-CLL Cells after Treatment with Recombinant Tumor Necrosis Factor-Related Apoptosis-Inducing Ligand and Histone Deacetylase Inhibitors

**DOI:** 10.1155/2009/408038

**Published:** 2009-06-14

**Authors:** Lyse A. Norian, Tamara A. Kucaba, James K. Earel, Tina Knutson, Rebecca L. vanOosten, Thomas S. Griffith

**Affiliations:** Department of Urology and Interdisciplinary Graduate Program in Immunology, University of Iowa Carver College of Medicine, Iowa City, IA 52242, USA

## Abstract

Tumor necrosis factor-related apoptosis-inducing ligand (TRAIL) is currently being investigated as a therapeutic agent for a variety of malignancies, as it triggers apoptosis specifically in transformed cells. However, TRAIL use as a stand alone therapeutic is hampered by the fact that many primary tumor cells are resistant to TRAIL-mediated apoptosis. Here, we investigated the extent to which pretreatment of TRAIL-resistant primary B-cell chronic lymphocytic leukemia (B-CLL) cells with histone deacetylase inhibitors (HDACis) could render them susceptible to killing by TRAIL. We found that HDAC inhibition in B-CLL cells led to increased TRAIL receptor expression, increased caspase activation, decreased expression of antiapoptotic regulators such as Bcl-2, and ultimately, enhanced TRAIL-induced apoptosis. Importantly, untransformed peripheral blood mononuclear cells remained largely resistant to TRAIL, even in the presence of HDACis. These results suggest that combination therapies using HDAC inhibition and TRAIL could prove beneficial for the treatment of B-CLL.

## 1. Introduction

Inducing apoptosis in tumor cells is a promising therapeutic approach for the treatment of both solid and lymphatic tumors. The TNF family member tumor necrosis factor-related apoptosis-inducing ligand (TRAIL) is an apoptotic mediator that has received much attention, due to its ability to bring about cell death specifically in tumor cells without affecting untransformed cells or tissues [1–3]. In humans, TRAIL interaction with either of two death-domain containing receptors, TRAIL-R1 (DR4) or TRAIL-R2 (DR5), leads to apoptosis in target cells [4–7]. Death receptor ligation activates the extrinsic apoptosis pathway and results in recruitment of FADD and caspase 8 to the death-inducing signaling complex (DISC), followed by activation of caspase 8 and caspase 3 [8–12]. This type of apoptosis differs mechanistically from that brought about by the cell intrinsic pathway, which is mediated by early activation of caspase 9, and leads to loss of mitochondrial membrane potential [13, 14]. Both TRAIL-R1 and -R2 have been found on a wide variety of primary tumor cells and tumor cell lines, making TRAIL-induced apoptosis ideal for therapeutic intervention in a number of malignancies. Equally important, recombinant soluble TRAIL protein has shown no toxicity in Phase I clinical trials [15] highlighting its safety as a therapeutic agent. Despite this potential, TRAIL use as a stand-alone therapeutic has been hampered by the fact that many primary tumor cells are inherently resistant to TRAIL-mediated apoptosis, despite expressing extracellular TRAIL receptors [16].

B-cell chronic lymphocytic leukemia (B-CLL) is the most frequent type of leukemia found in western countries [17]. It is characterized by an accumulation of mature, nonproliferating B lymphocytes in the blood, spleen, lymph nodes and bone marrow. The accumulation of B-cells results not from a proliferative defect, but rather from a failure of B-cells to undergo apoptosis, leading to large numbers of cells blocked in the *G*_0_/*G*_1_ phase of the cell cycle [18]. Not surprisingly, then, prior studies have shown that B-CLL cells are resistant to TRAIL-induced apoptosis [19]. The reasons for this are unclear, but may include a combination of the following: reduced expression of the death-inducing receptors TRAIL -R1 and -R2, reduced expression of downstream caspase 8, or overexpression of antiapoptotic molecules such as FLIP. To circumvent this inherent resistance to TRAIL-mediated apoptosis, we sought to determine the extent to which using TRAIL in combination with histone deacetylase inhibitor (HDACi) administration could bring about apoptosis in primary B-CLL cells.

HDACi are a promising group of anticancer agents that have been used successfully alone or in combination to induce tumor cell death in vitro and in vivo [20, 21]. HDACi function by regulating chromatin structure, leading to the transcription of genes that are normally repressed during tumor outgrowth. In normal cells, chromatin structure is tightly regulated by the contradictory actions of two enzymes. Histone acetyltransferases (HATs) add acetyl groups to lysine residues on histone tails that protrude from compacted nucleosome structures [22]. This leads to a relaxation and an opening of the chromatin structure, allowing transcription factors to bind, and gene transcription to occur. HAT function is counteracted by HDACs, enzymes that remove acetyl groups, thereby causing a recompaction of the nucleosomes, and a silencing of gene transcription. By preventing histone deacetylation, HDACi maintain chromatin in an open structure [23]. Because many tumors overexpress HDACs, normal transcription of genes, including tumor suppressor genes, is suppressed. Treatment of tumor cells with HDACis counteracts this abnormality and results in a net inhibition of tumor cell cycle progression, and induction of either or both of the intrinsic and extrinsic apoptotic pathways, depending on the type of tumor cell being studied. HDACis also have effects that are independent of histone de-acetylation, including acetylation of nonhistone proteins such as p53, Rb, and hsp90, induction of p21 with consequent cell cycle arrest, and generation of oxidative stress via production of reactive oxygen species [24]. At present, 11 different HDACs have been identified, grouped into 4 families, and HDACi may target one or more of these molecules, depending upon their structure [24]. Additionally, HDACis have proven to be nontoxic to untransformed cells, leading to their recent use in a number of clinical trials.

Based on previous studies by our group and others, in which the combination of HDACi and TRAIL was able to bring about apoptosis of tumor cells that were resistant to single therapy with either agent alone [25–30], we sought to examine the use of HDACi and TRAIL as a potential combination therapy for B-CLL. We examined a panel of HDACi for their ability to upregulate TRAIL-R1 and -R2 on B-CLL cells from cancer patients, and to sensitize those cells to TRAIL-mediated apoptosis. We found that use of either Oxamflatin (Oxam) or Trichostatin A (TSA), two hydroxamic acid-based HDACis, sensitized B-CLL cells to TRAIL-mediated apoptosis, due to their ability to upregulate both TRAIL-R1 and TRAIL-R2, increase activation of caspase 8, and increase expression of the proapoptotic protein Bax while decreasing expression of the antiapoptotic molecules Bcl-x_L_ and Bcl-2. Importantly, the combination therapies showed limited toxicity against normal PBMC. Thus, our study illustrates the feasibility of using HDACi and TRAIL in combination to mediate apoptosis of B-CLL cells.

## 2. Materials and Methods

### 2.1. Reagents and Antibodies

The HDACi Trichostatin A (resuspended in EtOH) was purchased from Sigma (St. Louis, MO, USA); Oxamflatin (resuspended in DMSO) was purchased from Calbiochem (San Diego, CA, USA). All other HDACis are as previously described [30]. Antibodies against TRAIL-R1 and TRAIL-R2 (eBioscience, San Diego, CA, USA) were used for flow cytometric analysis according to the manufacturer's suggestions. Recombinant human TRAIL (Peprotech, Rocky Hill, NC, USA) consisted of the 168 amino acid extracellular domain, and was used at the indicated doses.

### 2.2. Primary Cell Preparations

Peripheral blood samples were obtained from patients at the University of Iowa Holden Comprehensive Cancer Center who were diagnosed with B-CLL, or from healthy control donors. Experiments were repeated 3–5 times, using cells from different donors. Lymphocytes were enriched by density gradient centrifugation using Ficoll-Hypaque. Cells were cultured in RPMI 1640 supplemented with 10% FCS, 1% nonessential amino acids, 1 mM sodium pyruvate, and 1% penicillin/streptomycin solution. This study was conducted according to protocols approved by the institutional review board at the University of Iowa, and informed consent was obtained from all patients before blood sample collection.

### 2.3. In Vitro Killing Assay

Cells were cultured in complete medium at 2 × 10^4^ cells/well in 96-well plates. Cells were either cultured in medium alone or in the presence of individual HDACi at the indicated doses, for 16 hours. After this pretreatment, recombinant human TRAIL was added at 100 ng/mL, and cells were cultured for an additional 4 hours. Cell death was determined by costaining for Annexin V and propidium iodide, to permit identification of apoptotic versus necrotic cells.

### 2.4. Quantitation of Caspase Activity and Mitochondrial Membrane Potential

Briefly, cells were cultured in complete medium, in the presence or absence of individual HDACi for 16 hours, followed by a 4 hour treatment with TRAIL as above. Caspase activity was quantitated as described previously [30]. Briefly, cells were harvested, and incubated with fluorescently labeled caspase inhibitor peptides, according to the manufacturer's protocol (Immunochemistry Technologies, Bloomington, MN, USA). Inhibitor peptides were as follows: Caspase 3 = carboxyfluorescein- Asp-Glu-Val-Asp; Caspase 8 = carboxyfluorescein- Leu-Glu-Thr-Asp; Caspase 9 = carboxyfluorescein- Leu-Glu-His-Asp. MMP was quantitated via the use of the MitoPT mitochondrial permeability transition kit according to the manufacturer's suggested protocol (Immunochemistry Technologies, Bloomington, MN, USA).

### 2.5. Quantitative Reverse Transcription PCR

Total RNA was isolated from cultured cells with Trizol reagent (Invitrogen, Carlsbad, CA, USA) according to the manufacturer's protocol. Reverse transcription of total RNA was done by using Superscript II. The real-time qRT-PCR primer and probe sets were purchased from Applied Biosystems (Foster City, CA). cDNA (250 ng) was used as a template for TaqMan Assays and the internal control of rRNA. The TaqMan reaction was conducted as previously described [31].

## 3. Results

### 3.1. The HDACi Oxam and TSA Upregulate TRAIL Receptor Expression on B-CLL Cells

**Figure 1:**
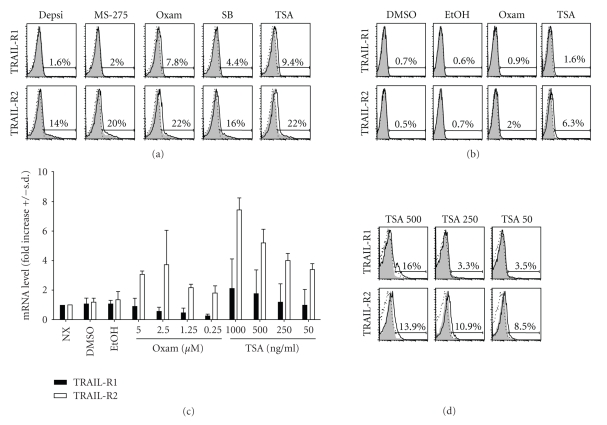
*HDAC inhibition by Oxam and TSA leads to TRAIL receptor expression on B-CLL cells*. (a) The percentages of B-CLL cells expressing TRAIL-R1 or TRAIL-R2 are shown. B-CLL cells were cultured for 16 hours in the presence or absence of individual HDACi, as indicated, then analyzed by flow cytometry for TRAIL-R1 and TRAIL-R2 surface expression. Filled grey histogram = Isotype control, dotted line = untreated, solid line = HDACi. (b) Enriched PBMC from healthy donors were cultured for 16 hours as indicated, then the surface expression levels of TRAIL-R1 and TRAIL-R2 were analyzed by flow cytometry. Filled histogram = Isotype control, dotted line = untreated, solid line = vehicle alone or HDACi. Numbers indicate the percentages of positively staining cells, compared to isotype. (c) TRAIL-R1 and TRAIL-R2 mRNA levels in B-CLL cultured as indicated for 16 hours. (d) The percentages of B-CLL cells expressing surface TRAIL-R1 or TRAIL-R2 in response to varying doses of TSA are given. Filled histogram = Isotype control, dotted line = untreated, solid line = vehicle alone or HDACi. For all panels, one experiment, representative of at least 5 experiments using cells from different donors, is shown.

To evaluate the potential therapeutic efficacy of combining HDACi with recombinant TRAIL for the treatment of B-CLL, we began by examining the ability of a panel of HDACi to upregulate TRAIL receptor expression on B-CLL. Human B-CLL were cultured for 16 hours in the presence or absence of individual HDACi. As shown in Figure 1(a), untreated B-CLL express little to no TRAIL-R1 or TRAIL-R2. In contrast, several HDACis, including Oxam and TSA, were able to upregulate expression of both TRAIL-R1 and TRAIL-R2 on B-CLL, although the increases in TRAIL-R1 were modest compared to those observed for TRAIL-R2. Not all HDACis were equivalent in their ability to upregulate TRAIL receptor expression, as MS-275 and DEPSI had little effect on TRAIL-R1 expression, yet strongly upregulated TRAIL-R2 (Figure 1(a)). 

Because we have shown previously that combination therapy with TRAIL and HDACi can lead to TRAIL sensitivity in untransformed cells [32], it was critical to determine the extent to which Oxam or TSA administration would lead to TRAIL-R1 and -R2 upregulation on normal PBMC. PBMC were obtained from healthy donors, and were incubated with Oxam or TSA for 16 hours. After this time, cells were harvested and analyzed by flow cytometry to assess whether TRAIL-R1 and/or TRAIL-R2 surface proteins were upregulated. Untreated PBMC from healthy donors did not express TRAIL -R1 or -R2, and neither Oxam nor TSA caused increased expression of these proteins (Figure 1(b)).

To assess whether the upregulated surface expression of TRAIL-R1 and TRAIL-R2 on B-CLL cells was due to changes in the quantity of mRNA present, we cultured cells for 16 hours in the absence or presence of varying doses of Oxam or TSA. Both Oxam and TSA were found to increase mRNA levels of TRAIL- R2 (Figure 1(c)), suggesting that these compounds were acting to promote gene transcription. In contrast, Oxam did not elevate TRAIL-R1 mRNA levels over untreated controls, indicating that a transcription-independent mechanism was responsible for the upregulation of TRAIL-R1 surface expression. Some experiments actually showed a decrease in TRAIL-R1 mRNA levels in the presence of Oxam versus untreated controls, which could suggest that Oxam was decreasing transcription or altering the half-life of TRAIL-R1 mRNA in those instances. While the 500 ng/mL dose of TSA led to clear enhancement of TRAIL-R1 and -R2 protein expression, lower doses failed to reproducibly affect TRAIL-R1 surface expression (Figure 1(d)). Therefore, specific doses of 2.5 uM Oxam and 500 ng/mL TSA were used for all subsequent experiments. Collectively, these results show that Oxam and TSA induce transformed B-CLL, but not normal PBMC, to upregulate TRAIL-R1 and TRAIL-R2 surface expression, through transcription-dependent and independent mechanisms.

### 3.2. Treatment with HDACi Sensitizes B-CLL Cells to TRAIL-Induced Apoptosis

**Figure 2:**
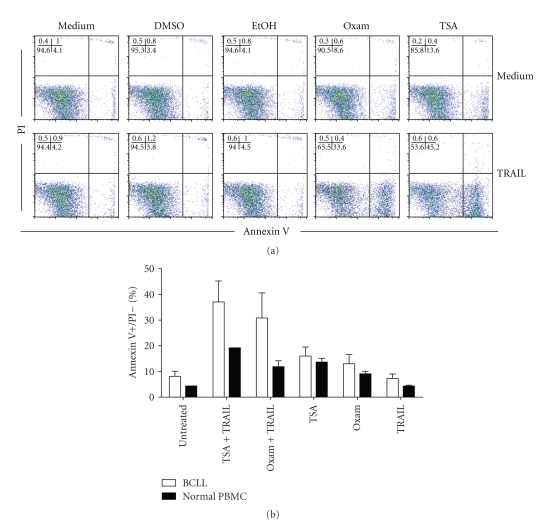
*HDACi treatment sensitizes B-CLL cells to TRAIL-induced apoptosis*. (a) B-CLL cells were cultured with or without 2.5 *μ*M Oxam or 500 ng/ml TSA or vehicle alone for 16 hours before addition of 100 ng/ml rhTRAIL. Cultures were harvested 4 hours later, and the percentage of cells undergoing apoptosis was determined by Annexin V staining. One experiment, representative of more than 5 experiments using cells from different donors, is shown. (b) The mean percentage of Annexin V^+^/PI^−^ cells is shown for each treatment group. Cells were cultured as in (a).

Once we had determined that Oxam and TSA upregulated TRAIL receptor expression on B-CLL cells, we then determined whether this translated into an increase in susceptibility to TRAIL-mediated apoptosis. B-CLL cells were again cultured alone (untreated) or in the presence of 2.5 *μ*M Oxam or 500 ng/mL TSA for 16 hours prior to the addition of recombinant TRAIL at 100 ng/ml. Cultures were harvested 4 hours later, and the percentage of TRAIL-induced apoptosis measured by Annexin V staining. As controls, normal PBMCs were cultured simultaneously. In agreement with previous reports [19, 33, 34], we found that untreated B-CLL were resistant to killing by TRAIL alone (Figures 2(a) and 2(b), <10% cell death). The addition of either Oxam or TSA in the absence of TRAIL caused only minimal increases in B-CLL apoptosis (mean = 15% and 18%, resp.). In contrast, when recombinant TRAIL was added after either Oxam or TSA pretreatment, roughly a third of B-CLL cells became apoptotic within the 4 hours TRAIL incubation period. Importantly, PBMC from healthy donors were much less susceptible to TRAIL-mediated killing, even in after pre-incubation with Oxam or TSA (mean = 18% and 12%, resp.). The majority of the cell death observed in PBMC was due to the actions of TSA or Oxam, rather than the addition of TRAIL (compare PBMCs with TSA alone versus TSA + TRAIL). To determine whether longer incubations with HDACi alone would cause greater percentages of cells to undergo apoptosis, we cultured B-CLL cells or PBMC alone or in the presence of Oxam or TSA for 24 or 48 hours. However, the percentages of cells undergoing apoptosis remained similar to those shown in Figure 2(b) (data not shown). Therefore, the HDACi Oxam and TSA preferentially sensitize B-CLL to TRAIL-mediated apoptosis, as compared to normal PBMCs.

### 3.3. TRAIL-Mediated Caspase Activation Is Enhanced by the Presence of HDACi

**Figure 3:**
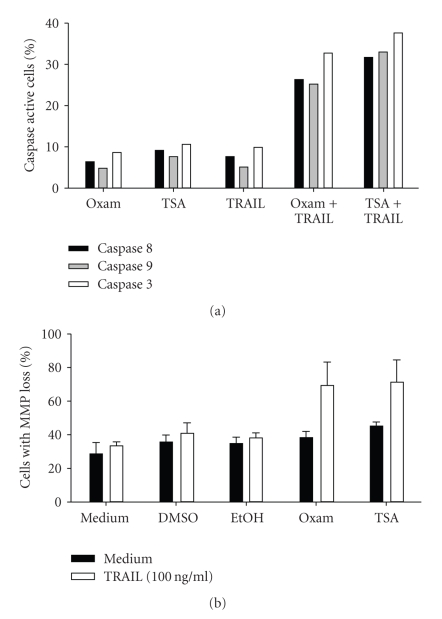
*HDACi pretreatment leads to increased caspase activity and MMP loss with TRAIL administration*. (a) B-CLL cells were cultured in the presence or absence of 2.5 *μ*M Oxam or 500 ng/ml TSA for 16 hours, followed by a 6 hours incubation with rhTRAIL (100 ng/ml). At that time, cells were harvested, and their caspase activity was determined as described in the Methods. (b) B-CLL cells were cultured in the presence or absence of 2.5 *μ*M Oxam or 500 ng/ml TSA for 16 hours, followed by a 6 hour incubation with or without rhTRAIL at 100 ng/ml. Mitochondrial membrane potential was assayed as described in the Methods. One experiment each, representative of more than 5 experiments using cells from different donors, is shown.

We next wanted to determine the mechanism by which Oxam and TSA sensitized B-CLL cells to TRAIL-mediated killing. TRAIL induces apoptosis via activation of downstream caspases, including caspase 8, and this signal can be augmented by the loss of mitochondrial membrane potential (MMP). B-CLL cells were again cultured in the presence or absence of either Oxam or TSA for approximately 16 hours, followed by culture with recombinant TRAIL. Preculture of cells with Oxam or TSA led to striking increases in the activity of caspases 8, 9, and 3 after TRAIL administration (Figure 3(a)). As expected, the activity of all three caspases remained low when Oxam, TSA or TRAIL were used individually.

Both capsases 8 and 3 are activated during the extrinsic apoptosis pathway, whereas caspase 9 is associated with the intrinsic apoptosis pathway [35]. Triggering of the intrinsic pathway leads to a loss of MMP and release of cytochrome C, which allows cytochrome C to then interact with Apaf 1 and procaspase 9, eventually resulting in active caspase 9 [36]. Because caspase 9 activity was increased after B-CLL treatment with HDACi and TRAIL, this suggested that cells were also experiencing a loss of MMP. In fact, when we examined B-CLL for a loss of MMP, we found that pretreatment with HDACi followed by TRAIL administration increased the percentage of cells exhibiting a loss in MMP (Figure 3(b)). This was not observed in B-CLL treated with either HDACi or TRAIL alone. Thus, both the extrinsic and intrinsic apoptotic pathways are initiated in B-CLL cells treated with the combination of HDACi and TRAIL. 

**Figure 4:**
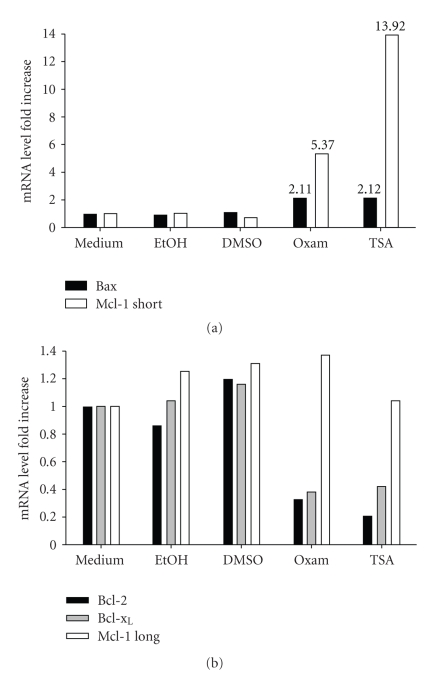
*HDAC inhibition prior to TRAIL administration primes cells for death via alterations in levels of apoptotic regulators*. B-CLL cells were cultured with or without 2.5 *μ*M Oxam or 500 ng/ml TSA for 16 hours prior to cell harvest and RNA preparation. (a) The fold change in mRNA levels for the indicated pro-apoptotic regulators is shown. (b) The fold change in mRNA levels for the indicated antiapoptotic regulators is shown. One experiment each, representative of more than 5 experiments using cells from different donors, is shown.

The preceding experiments indicated that HDACi pretreatment sensitized B-CLL cells to subsequent apoptosis with TRAIL. Mechanistically, this appeared to result from a variety of factors, including enhanced TRAIL receptor expression, increased caspase activation, and loss of MMP. Additional mechanisms of sensitization could also include activation of pro-apoptotic regulators and inhibition of antiapoptotic molecules. To determine if these factors were also contributing to elevated TRAIL-mediated apoptosis following HDACi treatment, we examined changes in apoptotic regulators at the mRNA level. As before, B-CLL cells were treated with either Oxam or TSA for 16 hours, then examined for changes in mRNA levels of several key apoptotic regulators. Both Oxam and TSA led to modest increases in mRNA for the pro-apoptotic molecule Bax, but substantial increases for the pro-apoptotic molecule Mcl-1 short, resulting in a 13-fold increase in mRNA levels with TSA treatment (Figure 4(a)). At the same time, both Oxam and TSA produced sharp decreases in mRNA levels for the antiapoptotic molecules Bcl-2 and Bcl-x_L_ (Figure 4(b)). Therefore, the HDACi Oxam and TSA sensitized B-CLL cells to TRAIL-mediated apoptosis by both increasing levels of pro-apoptotic regulators and decreasing levels of molecules that normally protect cells from undergoing apoptosis.

## 4. Discussion

B-CLL is the most prevalent form of adult leukemia, and is characterized by an accumulation of mature, often CD52^+^ B lymphocytes in the bone marrow and blood [17, 18]. At present, treatment options for B-CLL are limited, and progressive disease is typically fatal. Campath (alemtuzumab) is an anti-CD52 monoclonal that targets B-CLL cells, and is currently one of the more promising therapeutic options, although response rates remain at less than 40% [37]. Clearly, new treatment options are needed for these patients.

TRAIL is a promising anticancer agent, given that it can specifically induce apoptosis in transformed cells, without affecting normal host cells or tissues [1–3]. A major limitation of TRAIL therapy is the fact that many primary tumor cells are inherently resistant to TRAIL-mediated killing [16]. The reasons for this vary, but may include a lack of expression of TRAIL receptors on tumor cells, inadequate caspase activation after TRAIL receptor ligation, or over-expression of antiapoptotic molecules such as Bcl-2. This fact has led to the pursuit of TRAIL-based combination therapies, in which TRAIL administration is combined with other anticancer agents in an attempt to render tumor cells susceptible to TRAIL-mediated killing.

We and others have previously explored the use of TRAIL in conjunction with HDACi administration [25–30]. HDACis are also being pursued as anticancer therapeutics, given that they can bring about tumor cell apoptosis by reversing transcriptional repression. Because the modes of action of TRAIL and HDACi are distinct, their combined use appears to lead to synergistic killing of many tumor types that are normally TRAIL resistant, including bladder cancer cells, renal cell carcinomas, and prostate cancer cells [25–30]. For example, although TRAIL and HDACi combination therapies were shown to be successful in bringing about apoptosis in renal cell carcinoma lines, we also observed that combination therapies caused untransformed human renal proximal tubule epithelial cells to become susceptible to TRAIL-mediated killing [32]. Therefore, the combined use of two agents which by themselves do not kill untransformed cells led to apoptosis of normal cells. This underscores the importance of evaluating potential TRAIL-based combination therapies for their effects on untransformed primary tissues. Despite this fact, TRAIL remains one of the most promising anticancer biologics, given that phase I clinical trials with TRAIL alone demonstrated no toxicity [15] and numerous preclinical studies have shown that TRAIL as a stand-alone agent does not target normal cells. Thus, the challenge is to find doses and combinations of agents that render TRAIL- resistant tumor cells susceptible to TRAIL-mediated killing, while sparing normal host tissues from toxic side effects.

In this study, we found that pretreatment of primary B-CLL cells with either of two HDACis, Oxam, or TSA, led to upregulated TRAIL receptor expression, increased caspase 8 activation, triggered a loss of MMP, and decreased expression of antiapoptotic regulators such as Bcl-2. The net result was a significant increase in TRAIL-mediated killing of B-CLL cells, such that over 30% of B-CLL cells became Annexin-V^+^ during the 4 hours TRAIL incubation period. This is in comparison to B-CLL cells treated with TRAIL alone, which produced apoptosis in fewer than 10% of cells. This reversal in susceptibility to TRAIL-mediated apoptosis indicates the efficacy of B-CLL pretreatment with HDACi. Importantly, normal PBMCs were much more resistant to the combination of HDACi + TRAIL, suggesting that this type of therapy may have applicability in B-CLL patients. The combination of Oxam + TRAIL produced less apoptosis in normal PBMC than did TSA + TRAIL, which indicates that it may be possible to modify the timecourse or dose of agents in vivo to further limit killing of untransformed cells. 

Our observations extend work by Garofalo et al. in which they examined the role of PED, an antiapoptotic molecule, as a mechanism for B-CLL resistance to TRAIL-mediated killing [33]. In that study, the authors found that B-CLL cells treated with PED antisense oligonucleotides, protein synthesis inhibitors, or the HDACi valproic acid or TSA could downregulate PED expression, rendering B-CLL cells susceptible to TRAIL killing. Interestingly, the authors concluded that treatment of B-CLL cells with TSA did not alter the expression of other antiapoptotic molecules such as Bcl-2, nor did it increase expression levels of TRAIL-R1 and TRAIL-R2. In contrast, we find that a five-fold higher dose of TSA (500 ng/ml) does lead to increased mRNA levels for TRAIL-R1 and TRAIL-R2, does upregulate surface expression of these same receptors, and does cause Bcl-2 mRNA levels to decrease substantially. These findings highlight the importance of dose effects, even with the same anticancer agents, and reveal that HDAC inhibition by TSA affects multiple pathways that culminate in B-CLL sensitivity to TRAIL-mediated apoptosis.

## 5. Conclusions

We show here that pretreatment of TRAIL resistant B-CLL cells with either of two HDACis, Oxam, or TSA, renders cells susceptible to subsequent killing by recombinant TRAIL protein. Both Oxam and TSA led to increased surface expression of TRAIL-R1 and TRAIL-R2, increased caspase 8 and caspase 3 activation, a loss of MMP, and changes in mRNA levels for key pro- and antiapoptotic regulators, all of which combined to sensitize B-CLL cells to TRAIL-induced apoptosis. Importantly, combination therapies preferentially sensitized transformed cells to apoptosis, as the same level of killing was not observed in normal PBMC. Thus, combination therapies using specific HDACi and recombinant TRAIL may prove to be efficacious for the treatment of B-CLL in humans.
